# A Layman's Account of a Ward's Equipment

**Published:** 1915-12-18

**Authors:** 


### A LAYMAN'S ACCOUNT OF A WARD'S EQUIPMENT.

### What He Found at University College Hospital.

Efficiency does not necessarily mean great expense any more than a lavish expenditure of money can be taken as a guarantee of efficiency. In these days, when the highest and the lowest are alike beginning to grasp the necessity of saving, it is well, perhaps, to remember that efficiency and true economy are synonymous terms, but that parsimony will destroy both. In no phase of work is this fact capable of greater demonstration than in the administration of our hospitals.

To-day, more perhaps than at any other period, the object of boards of management is to secure efficiency at the lowest possible cost. No one will deny that this is a laudable desire, and it is in order to contribute to the attainment of this end that we have decided to publish a series of articles on hospital equipment.

Experience, and the information gained by an exchange of ideas, are valuable as a means towards efficient administration; and it is hoped that by illustrating types of equipment in different hospitals the articles may prove practical and suggestive.

### Bedside Furniture.

As an introductory article we give a description of part of the equipment of a surgical ward at the University College Hospital, Gower Street. This hospital, owing to the site, has been planned in the form of a Maltese Cross. The result aimed at is, that sunshine should find its way in fair proportion to each block of wards during each twenty-four hours. On entering a surgical ward, which has accommodation for twenty-four patients, we find each bed placed between two windows so that the occupants may have the best of air and light, whilst draughts are minimised by special fittings for the windows with safety catches. Over each bed is a pulley, chain and handle, within easy reach of the patient, so that he may be able to raise himself, and on the side of each bed is a locker and a chair. The locker serves various purposes. It comprises a cupboard, or box, which may be opened at the front or on the top, and there are also two small shelves. The locker is provided with a wooden flap, which can be raised to the desired height and converted into a table with movable supports. The locker runs smoothly on wheels, and can be readily placed in position near the bed for convenient use by the patient. Against an upright wooden back is suspended the chart, which may be transferred to the back at any time, where there is also a rail for a towel. Each bedstead in the ward runs freely on wheels, and iron heads can be instantly lowered so that the maximum facilities are readily afforded for dressing every patient.

### Patients' Chairs and Trolleys.

The ward furniture includes two large tables, each having a form on either side, two couches, two armchairs, a weighing machine, and four chairs on wheels, in which patients can move themselves about the ward. A polished parquetry floor contributes to their easy movement. Patients able to be out of bed have their meals served at one of the large tables. The reclining-couch can be adjusted to any position most comfortable for a patient. A desk and chair used by the sister are situated near the centre of the ward, and are so placed that she may have all the patients in sight. Each surgical ward has two, and sometimes three, dressing-trolleys. They are fitted with two metal trays which are movable and can readily be changed from one side to the other.

The ward dispensary on wheels, which is also of metal, occupies a position in the middle of the ward. The lower part is fitted with drawers, cupboards and shelves. Above these are two metal slabs with about a foot between each. The top one has a glass slab covering one end in order to prevent lotions from injuring the metal, and two small utensils supported by brass rods at one end, which prevent any lotion from dropping to the floor. A table with a marble slab is found a useful adjunct for glasses and other utensils.

On one side of the ward are the sterilising appliances and opposite is the test stand, above which is a metal cupboard, with a glass front and shelves, containing the testing apparatus. There is also an oxygen stand on wheels. Two wash-basins for the surgeons and two for the dressers or nurses, with nail-brushes and bowls containing lotions for the hands, are also provided, together with a stop-tap. There are eight three-fold screens.

### The Sanitary Unit.

At the extreme end of the ward is a door opening to the bathroom and lavatories, where space is provided for filling water-beds from two taps. An enamel bath with a thermometer is provided for testing the temperature, and an adjustable key for controlling the flow of water. Above the radiator is a rail for drying macintoshes and a shelf for wash-bowls and other utensils. Besides a row of pegs for dressing-gowns, iron cradles used in surgical cases also hang in the bathroom.

The corridor leading to the sanitary unit is provided with a series of lock-up cupboards for patients' outdoor clothes, bearing numbers corresponding to those of the beds in the ward. Below, two additional cupboards contain hot-water bottles and various cleansing appliances, and in the corridor is a pair of steps. Separate lavatories are provided for the patients and the staff. The doors of those for the patients are not provided with locks. The staff lavatory is fitted with a wash-basin, and beyond the lavatories is the general sink-room, with a hot and cold water supply for scrubbing macintoshes, etc., a rack for bed-pans, and a zinc-lined cupboard with three zinc shelves where specimens may be placed.

### The Ward Kitchen and Linen-room.

Another short corridor entered from the ward, and running parallel with the first, leads to the linen-room, which is provided with shelves and hanging curtains, and heated so that the clothes may be kept aired. The doors of the ward are on the swing principle, with suction-stops which prevent any noise arising from slamming. At the end of the corridor is a small ward kitchen, where breakfasts and teas for patients are prepared. Its furniture comprises a dresser, a lift-table, a gas-stove, and copper urn (hot water is always ready), a filter for drinking-water, an ice-box, and other kitchen utensils.

In addition to the electric globes which hang from the ceiling in the middle of the ward, there is an electric light over each bed. There are also various plugs in the walls of the ward to which, with the aid of a long coil of wire, a movable electric lamp can be fitted if required. Fixed to the large table just inside the entrance of the ward is a collection-box.

